# EEG microstate analysis of emotion regulation reveals no sequential processing of valence and emotional arousal

**DOI:** 10.1038/s41598-021-00731-7

**Published:** 2021-10-28

**Authors:** Josephine Zerna, Alexander Strobel, Christoph Scheffel

**Affiliations:** Faculty of Psychology, Technische Universität Dresden, Dresden, Germany

**Keywords:** Neuroscience, Psychology

## Abstract

In electroencephalography (EEG), microstates are distributions of activity across the scalp that persist for several tens of milliseconds before changing into a different pattern. Microstate analysis is a way of utilizing EEG as both temporal and spatial imaging tool, but has rarely been applied to task-based data. This study aimed to conceptually replicate microstate findings of valence and emotional arousal processing and investigate the effects of emotion regulation on microstates, using data of an EEG paradigm with 107 healthy adults who actively viewed emotional pictures, cognitively detached from them, or suppressed facial reactions. Within the first 600 ms after stimulus onset only the comparison of viewing positive and negative pictures yielded significant results, caused by different electrodes depending on the microstate. Since the microstates associated with more and less emotionally arousing pictures did not differ, sequential processing could not be replicated. When extending the analysis to 2000 ms after stimulus onset, differences were exclusive to the comparison of viewing and detaching from negative pictures. Intriguingly, we observed the novel phenomenon of a microstate difference that could not be attributed to single electrodes. This suggests that microstate analysis can detect differences beyond those detected by event-related potential analysis.

## Introduction

In neuroscience, a persevering division of imaging techniques based on their resolution has been holding its ground. Electroencephalography (EEG) is known for high temporal and poor spatial resolution while it is the other way around for functional magnetic resonance imaging (fMRI), so they are often combined^1,2^. However, methods for utilizing EEG as a spatial analysis tool have been around for years, like dipole source localization^3^ or the analysis of frequency bands^4^. Many of these methods are computationally demanding but there are more straightforward methods that have not been fully exhausted yet. One of these methods is microstate computation.

A microstate is a momentary distribution of activity across the scalp in EEG that persists for several tens of milliseconds before it changes into a different topographical distribution^5^. Microstates can be computed by clustering the topographical pattern of activity of each time point based on similarity^6^. Most studies focus on the duration and transition of microstates during rest, which differ in patients with neuropsychiatric diseases^7^. The majority of resting state studies compute microstates at time points of high global field power (GFP), a measure of the standard deviation of activity at a given time point^8^. This has been harshly criticized because it disregards less extreme topographies and the possibility of temporal overlap between microstates^9^.

A method somewhat contrary to microstate analysis is the analysis of event-related potentials (ERPs). An ERP is obtained by averaging the activity at a single or several adjacent electrodes within a certain time window across multiple trials to generate a continuous wave of voltage per time unit^10^. ERP components are activity peaks with a characteristic latency and polarity^11^ and are labelled accordingly, e.g. the P1 or P100 component for a positive peak 100 ms after stimulus onset^12^. Several emotion-related ERPs have been identified. Earlier ERPs within the first 200 ms are associated with perceptual processing such as stimulus valence^13,14^. Later components are associated with processes like emotional congruity^15^, elicited emotional arousal^16^, and response selection^17^, but also sensitive to stimulus valence^18,19^. Another ERP, the P300, has been shown to indicate an intrinsic salience of emotional stimuli^20^, even though it is quite late for a perceptual component. One key problem with focusing on single ERP components at single electrode sites is that of *cognitive subtraction*: one cannot simply assume that adding an aspect to a task will not change the operation of other aspects, and therefore it is not recommended to confine the comparison of two task versions to one anatomic site^21^. As a consequence, even in ERP research the impact of two experimental conditions on one component should be estimated by computing the magnitude of difference in scalp topography^11^.

Differences in scalp topography have scarcely been investigated in studies on emotion regulation (ER). ER refers to a variety of consciously or subconsciously applied strategies by which individuals influence not only the kind of emotion they experience but also its timing, subjective quality, and outward expression^22^. Two ER strategies that have been investigated most frequently are reappraisal, i.e. the cognitive transformation of an emotional stimulus through reinterpretation or detachment, and expressive suppression, the inhibition of emotional facial expressions^23,24^. There have been many fMRI studies demonstrating a top-down influence of prefrontal regions on the amygdala during ER^25–29^. EEG studies on ER either have a global focus on asymmetry of frequencies^30–32^ or a local focus on ERPs such as the Late Positive Potential (LPP), which peaks around 500 ms after stimulus onset at centro-parietal electrodes and can last for several seconds^18,33–37^. However, findings regarding the direction of influence of ER on ERP components are rather inconsistent. Results of the LPP depend on time window, recording site, age^38^, not on age but on up- or down-regulation in various ways^39–42^, and strategy^37^. Even the perceptual Early Posterior Negativity (EPN), which peaks between 200 and 300 ms after stimulus onset and indicates attention allocation^43^, appears to be either temporally shifted when watching negative stimuli^44^ or not affected by ER at all^41^.

To address these inconclusive ERP findings, Gianotti et al.^45^ used a microstate analysis on a paradigm with emotional stimuli, which allowed data-driven analysis without spatial or temporal confinement. They presented pictures that had very high or low values in valence or emotional arousal, respectively, while being balanced in the other quality. In the first 600 ms of viewing pictures with high (positive) and low (negative) valence, Gianotti et al.^45^ found microstate differences within 140–330 ms, while differences between pictures of high and low emotional arousal were observed at around 300 ms and after 520 ms. These temporal differences had not been identified by ERP studies before. Therefore, the authors concluded that valence information is being processed earlier than emotional arousal information.

The present study aimed to conceptually replicate the findings by Gianotti et al.^45^, applying their theoretic idea to a new context^46^. The context differed insofar that the participants were not naïve to the study’s theme of emotion and that the conditions also comprised ER strategies. Moreover, the aim is to investigate how the application of ER strategies becomes apparent in microstates, to further the understanding of ER and to advance microstate computation as an insightful analysis method for task-based paradigms. We therefore analyzed existing data of an ER block paradigm with EEG, in which healthy adults viewed, detached from, or suppressed their facial reaction to emotional pictures.

Our preregistered hypotheses (https://osf.io/9auv5/) were split into three parts: first, as a manipulation check, we expected lower subjective emotional arousal ratings after ER blocks than after blocks of active viewing, and that distinct EEG microstates can be computed within the first 600 ms of stimulus processing. Second, as a conceptual replication of Gianotti et al.^45^, we hypothesized that viewing emotional pictures would cause valence- and emotional arousal-based microstate differences. The former would emerge earlier and originate from different electrodes than the latter. Lastly, we expected microstate differences when comparing ER strategies for positive and negative pictures. Applying detachment would cause differences at later time points than expressive suppression, as studies comparing reappraisal and suppression have shown^37,47^.

## Results

Of the 106 participants with complete data sets, 49 chose Detachment and 57 chose Expressive Suppression in the Choice block. One participant’s data was missing the last block due to a power failure during recording. More than 60% of all 21,341 recorded trials were available for further analysis after artefact rejection (13,646 epochs, *M* = 127.53 per participant, *SD* = 43.31).

### Manipulation check

**Figure 1:**
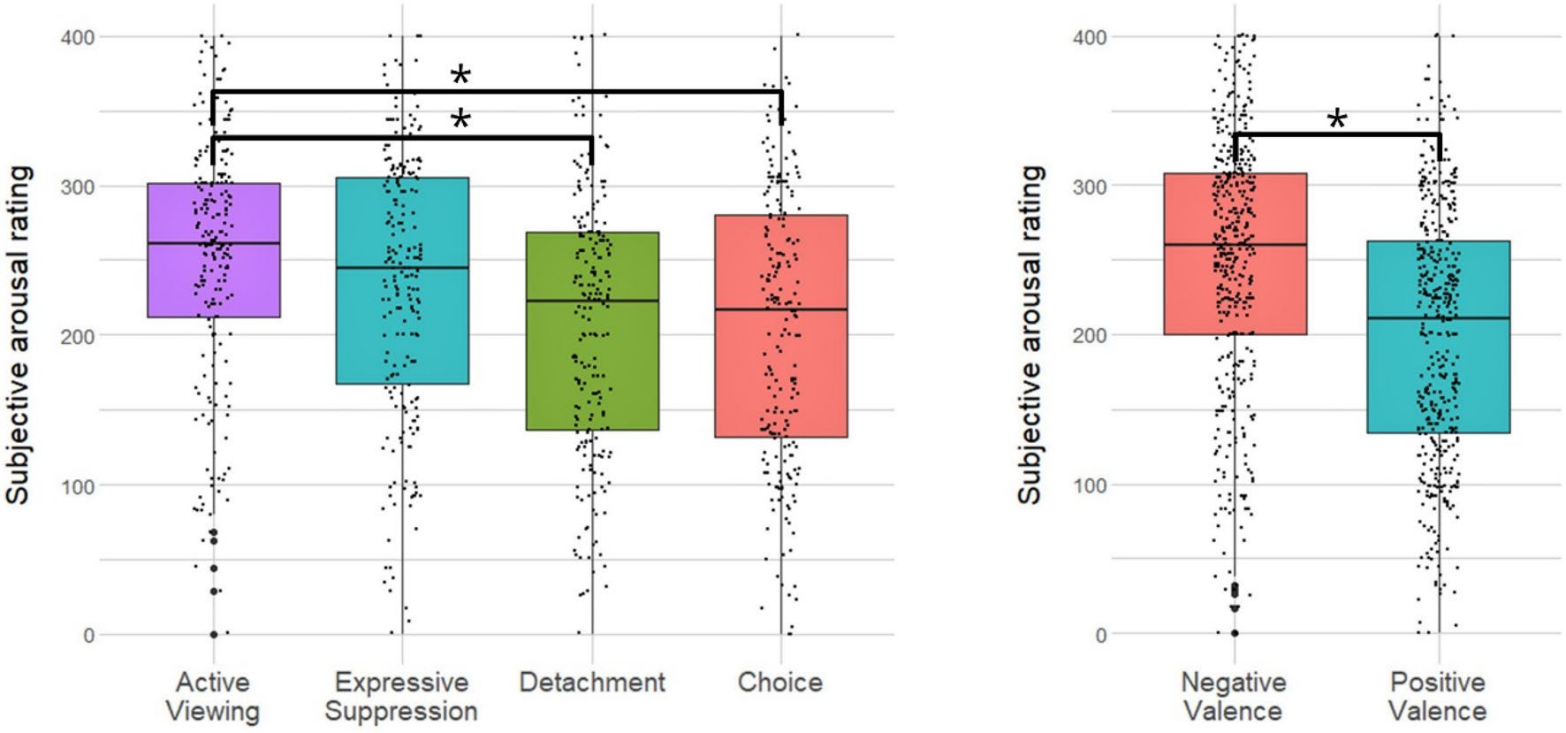
Change in subjective emotional arousal ratings depending on block and valence. Black horizontal lines within the box indicate the median, upper and lower box limits the upper and lower quartile, bold dots outside the whisker indicate outliers, i.e. 1.5 interquartile ranges from the box. Ratings of every participant are shown as a scatterplot along the whisker. Significant differences as computed with the Tukey’s Test are indicated by * (*p* < 0.001). Y-axis ticks were relabeled (“− 200” as “0”, “200” as “400”) for contentual logic.

The ANOVA showed a main effect of block, *F*(3, 848) = 15.6, *p* < 0.001, η_p_^2^ = 0.052, and valence, *F*(1, 848) = 70.9, *p* < 0.001, η_p_^2^ = 0.077, while the interaction of block and valence was not significant, *F*(3, 848) = 1.30, *p* = 0.27, η_p_^2^ = 0.005. Post-hoc analyses using Tukey’s Test indicated that subjective emotional arousal ratings were greater after the Active Viewing than after the Choice block, greater after the Active Viewing than after the Detachment block, and greater after negative than after positive pictures (*p* < 0.001, respectively) (Fig. 1).

### Microstate computation

Visual inspection of the *k*-means clustering options led to a choice of 18 microstates, formed by *k* = 14 clusters (Supplementary Table S2). The duration of the first and the last two microstates fell into the common range of a microstate in resting state studies (60–120 ms) but the remaining microstates fell below this duration. However, the clustering option that would have kept all microstates in the common range is *k* = 2, yielding six microstates. In this case, the resulting sum of inner cluster distance measures would have been disproportionately high compared to clusters with a larger *k*.

### Global topographical differences

**Figure 2:**
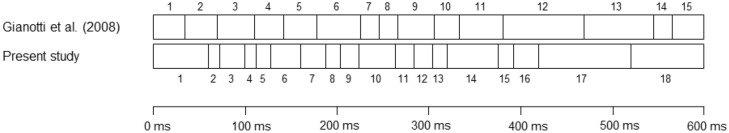
Temporal comparison of the microstates in both studies. Microstates as defined by the clustering of the grand–grandmean ERP. Cell length represents relative microstate length; numbers above or below denote the microstate number. Stimulus onset is at 0 ms.

When comparing the temporal characteristics of the 18 microstates here to the 15 microstates observed by Gianotti et al.^45^, it became apparent that there was only a small overlap (Fig. 2). In both studies, the microstates in the first 400 ms of the analysis window were shorter compared to the later microstates but start and end times were quite different. The one that was most similar was microstate 14 (here 320–376 ms after stimulus onset, in their study microstate 11, 330–378 ms after stimulus onset), while all other microstates showed little to no similarities.

**Figure 3:**
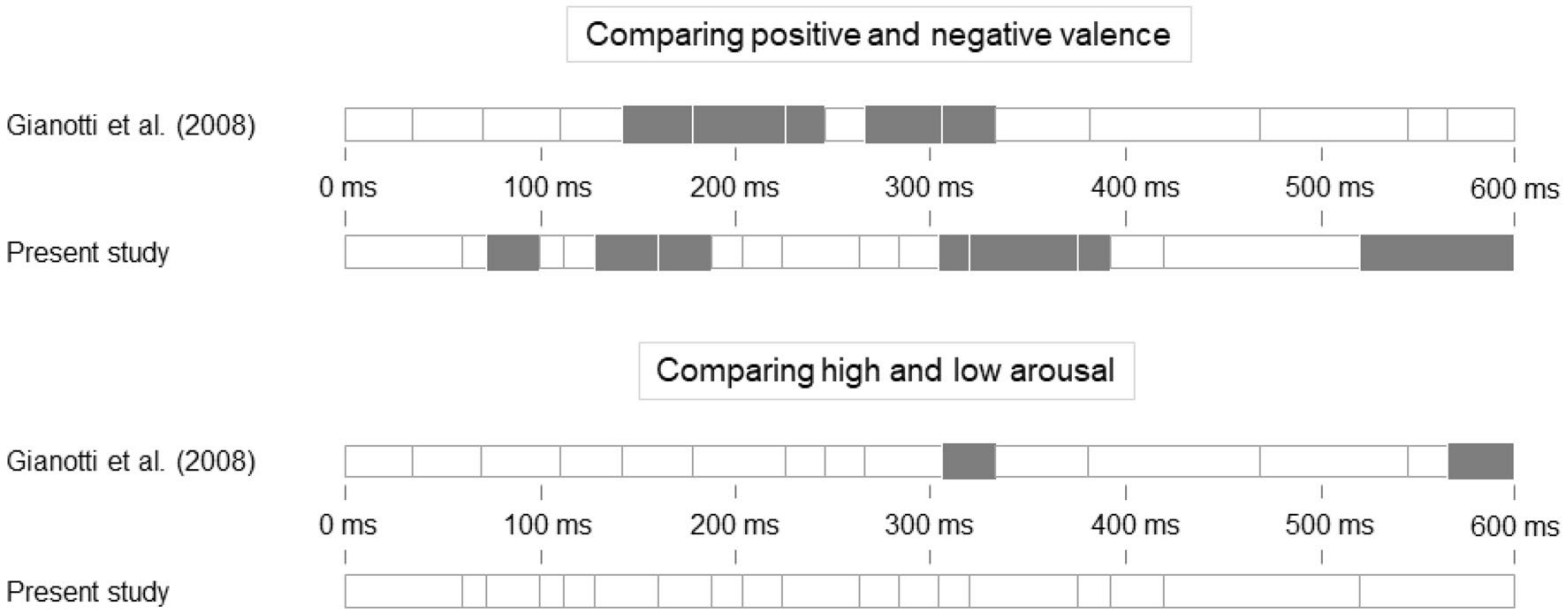
Side by side comparison of the significant microstates in the Active Viewing block of both studies. The upper panel shows the microstates with significant results in the TANOVA of negative and positive pictures in the Active Viewing block, the lower panel the ones of the TANOVA of highly and less emotionally arousing pictures in the Active Viewing block. Cell length represents the relative microstate length; colored cells are significant microstates. Stimulus onset is at 0 ms.

In the regular analysis, the TANOVA with the adjusted significance level of *α*_adj_ = 0.0028 yielded significant results in only one hypothesis, namely the comparison of positive and negative pictures in the Active Viewing block (Fig. 3). Seven out of the 18 microstate pairs were significantly different from each other (*cos* *θ*_MS3_ = 0.99, *cos* *θ*_MS6_ = 0.96, *cos* *θ*_MS7_ = 0.80, *cos* *θ*_MS13_ = 0.91, *cos* *θ*_MS14_ = 0.94, *cos* *θ*_MS15_ = 0.92, *cos* *θ*_MS18_ = 0.84, all *p* < 0.001). All of these microstates were on the lower end of their null distribution as indicated by the topographical vectors which were more different to each other than any of the 3000 topographical vectors of the randomly shuffled conditions. Higher similarity than that of the vectors in the null distribution would have computationally been possible as well, but it did not occur here.

**Figure 4:**
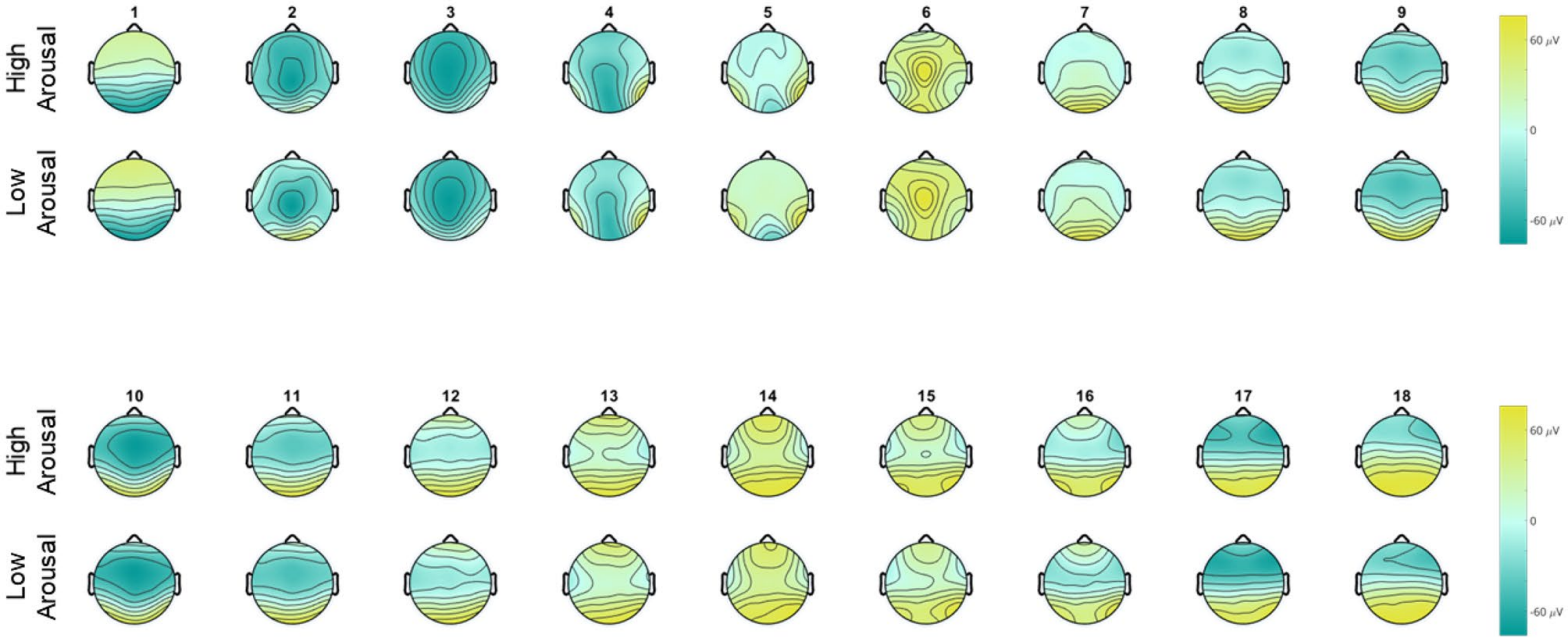
Microstates of actively viewing high and low emotional arousal pictures. Head seen from above, nose up. Microstates are numbered 1–18. No global differences emerged in the TANOVA, so no tests for local differences were conducted. Colors range from − 76 μV (teal) to + 76 μV (yellow).

We could confirm microstate differences within the Active Viewing block when comparing positive and negative valence. However, when comparing the results of the TANOVA for global topographical differences to those of Gianotti et al.^45^ (Fig. 3), there was only a 72 ms overlap between the significant microstates. No differences emerged in the emotional arousal-based comparison (Fig. 4).

The first overlap was from 142 to 188 ms after stimulus onset, shared by the microstates 6–7 of the present study and the microstates five to six of Gianotti et al.^45^. The second overlap was from 304 to 330 ms after stimulus onset, shared by the microstates 13–14 of the present study and the microstate ten of Gianotti et al.^45^. Almost half of the time covered by the significant microstates in the analysis by Gianotti et al.^45^ was between 200 and 300 ms after stimulus onset. This is a time window in which none of the microstates in the present study yielded a significant result.

In almost all twelve comparisons of conditions there was at least one microstate pair with *p* < 0.05, but not below the adjusted significance level. The only two exceptions were the comparison of Detachment Negative and Choice Negative, and the comparison of Expressive Suppression Positive and Choice Positive, which would not have yielded any significant results.

### Local topographical differences

**Figure 5:**
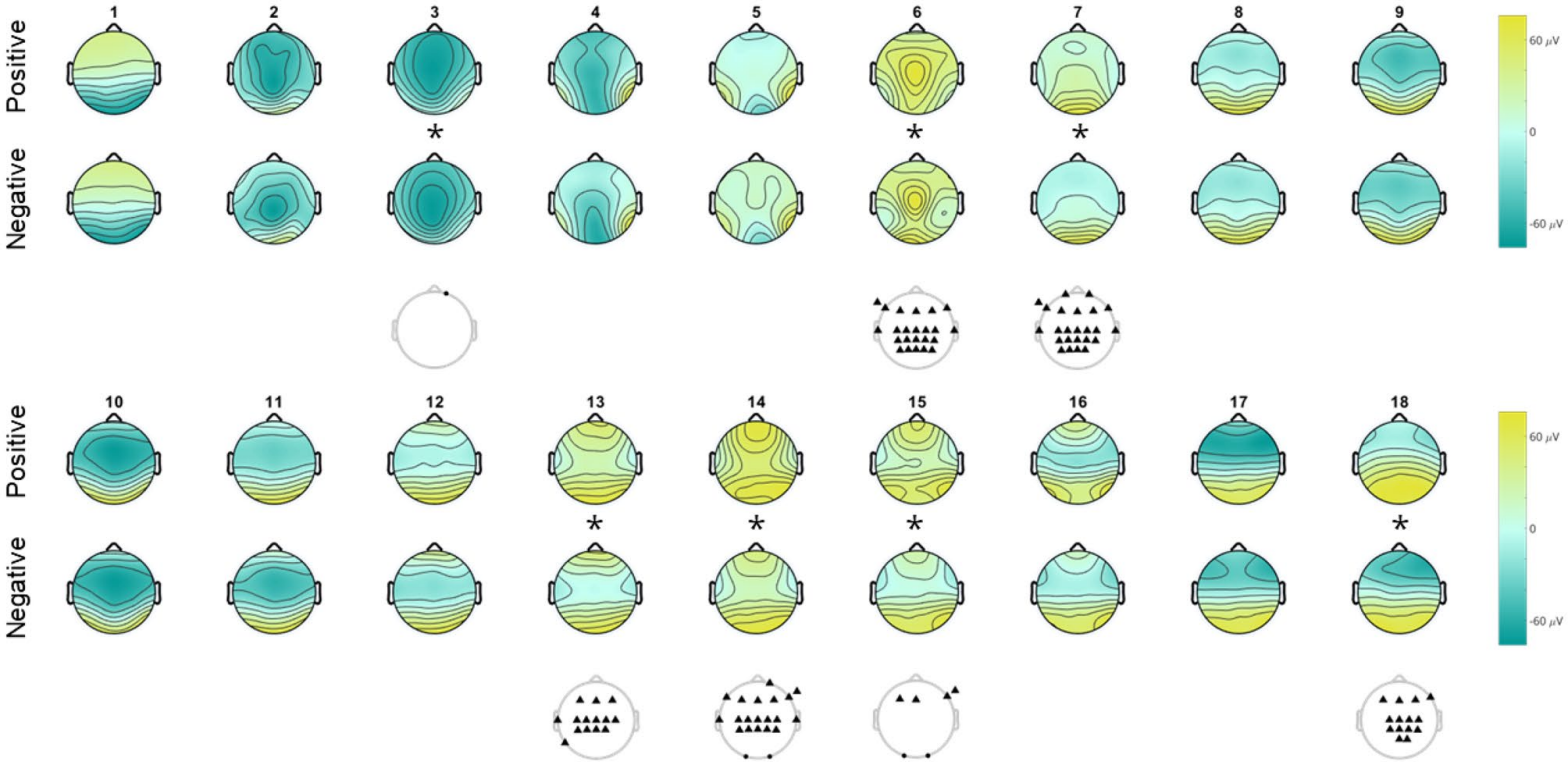
Global and local microstate differences between actively viewing positive and negative pictures. Head seen from above, nose up. Microstates are numbered 1–18. Significant global differences indicated by * (*p*_adj_ < 0.001). Electrodes with local differences are plotted below the microstate pair, ▲ indicates higher values in Active Viewing Positive, ● indicates lower values in Active Viewing Positive, all *p*_adj_ < 0.0017. Colors range from − 76 μV (teal) to + 76 μV (yellow).

Post-hoc analyses using two-sided paired-sample *t*-tests for the comparison of Active Viewing Positive and Active Viewing Negative showed that local differences strongly depended on the microstate (Fig. 5). In microstate three (72–100 ms after stimulus onset) there was only one channel with local differences, which was Fp2 with a smaller negative amplitude in the Active Viewing Negative condition. In microstates six and seven (128–188 ms after stimulus onset) the majority of frontopolar, frontal, temporal, central, centro-parietal, and parietal electrodes yielded significant results with higher positive amplitudes in the Active Viewing Positive condition. Microstate 13 (304–320 ms after stimulus onset) showed local differences in frontal, central, and centro-parietal electrodes along the midline and the left parietal lobe with higher positive amplitudes in the Active Viewing Positive condition. Microstate 14 (320–376 ms after stimulus onset) yielded many of the same central and centro-parietal as well as frontal and fronto-parietal electrodes in the right hemisphere with higher positive amplitudes in the Active Viewing Positive condition. Here, both occipital electrodes showed higher positive amplitudes in the Active Viewing Negative condition. Microstate 15 (376–392 ms after stimulus onset) showed the latter for both occipital electrodes, for a few frontal electrodes along the midline, and for the right temple. Lastly, microstate 18 (520–600 ms after stimulus onset) yielded significant results for dense groups of frontal, central, centro-parietal, and parietal electrodes along the midline with tendencies to the right hemisphere, all with higher negative amplitudes in the Active Viewing Negative condition. The *t*-test results for all seven microstates are listed in Supplementary Table S3.

### Exploratory analysis

The same preprocessing and analysis steps were applied to the time window of 600–2000 ms after stimulus onset. Visual inspection of the elbow plot led to the definition of 41 microstates formed by 14 clusters, therefore *p* was adjusted to *p*_adj_ = 0.0012. The duration of the microstates is listed in Supplementary Table S4. Again, only one hypothesis yielded significant results in the TANOVA, but in this case it was the comparison of viewing and detaching from negative pictures (see Supplementary Figure S6 for the topoplot). The three significant microstates were microstate two (644–668 ms after stimulus onset, *cos* *θ*_MS2_ = 0.78), microstate four (732–740 ms after stimulus onset, *cos* *θ*_MS4_ = 0.75), and microstate five (740–752 ms after stimulus onset, *cos* *θ*_MS5_ = 0.85, each at p < 0.001, respectively).

Local differences as identified by the two-sided paired-sample t-tests were confined to frontal and frontopolar electrodes in microstate two, and to all adjacent central, centro-parietal, and parietal electrodes at the crown of the head in microstate four, both with higher positive amplitudes in the Detachment Negative condition. In microstate five however, all electrodes exceeded the threshold of *p*_adj_ = 0.0017, resulting in a lack of local differences, even though the global difference of that microstate was statistically significant. This phenomenon had neither occurred in the regular analysis nor in the study by Gianotti et al.^45^. The t-test results for all three microstates are listed in Supplementary Table S5. In almost all twelve comparisons of conditions there was at least one microstate pair which was below the *p*-value of *p* = 0.05 but not the adjusted *p-*value. The only two exceptions were the comparison of Detachment Positive and Choice Positive, and the comparison of Expressive Suppression Positive and Choice Positive, which would not have yielded any significant results.

## Discussion

This study investigated the temporal and spatial characteristics of processing emotional pictures with different levels of valence and emotional arousal and how this is affected by emotion regulation. For that purpose, EEG microstates were computed using existing data of a block paradigm with healthy adults. We found global and local differences between microstates when comparing positive and negative stimulus valence in the Active Viewing block but did not find any differences when comparing high and low levels of emotional stimulus arousal. Therefore, we could not conceptually replicate the findings of a clear temporal structure of valence and emotional arousal processing by Gianotti et al.^45^. Effects of emotion regulation strategies were confined to the comparison of Active Viewing and Detachment of negative pictures, but only when the time window of analysis was extended in an exploratory approach.

### Conceptual replication of Gianotti et al.^45^

Cluster analysis yielded three more microstates than in Gianotti et al.^45^, and the start and end times differed considerably. Yet, both studies identified shorter microstates within the first 400 ms, reflecting the valence-independent, rapid processing of emotional stimuli^48–50^. Overall, we could not replicate the findings of Gianotti et al.^45^. And even though they stated that they corrected for multiple comparisons, their resulting *p*-value of 0.10 is still too high to counteract the inflation of false positives^51^. Therefore, although the present study was a conceptual replication, our more rigorous analysis suggests that there might indeed not be a sequential processing of valence and emotional arousal. The following section discusses the findings of the valence and emotional arousal-based analysis.

#### Implications of the comparison of positive and negative valence

The significant microstate pairs of viewing positive and negative pictures were spread across the 600 ms window, and only few share features with established ERP components. For instance, the first significant microstate (72–100 ms) might reflect an early P1 occurrence. This component is larger for negative stimuli at frontal, parietal, and occipital sites^13,14^, reflecting increased attention allocation^52^. Microstates six and seven, which spanned from 128 to 188 ms, include the beginning of ERP modulation by valence at 150 ms^53^, as well as the occipito-temporal N170, which is sensitive to faces and emotional expression^54,55^. A related component, the Late N1, has been shown to be habituation resistant to highly emotionally arousing, unpleasant stimuli^56^. In microstates six and seven, as well as in subsequent microstates, there were pronounced local differences over the parietal cortex, reflecting this region’s involvement in target processing^57^. The local differences over frontal areas might point to a very prompt evaluation of stimulus appetitiveness^58^. This illustrates how processing of emotional stimuli is not confined to posterior areas and how spatial restriction in ERP recordings can lead to an oversight in the time course of cognitive processing.

Surprisingly, there were no significant results in the 200–300 ms window, although multiple studies reported a valence-sensitive N2 component^13,14,59^ and ERP modulation by unpleasant pictures^60^. Microstates 13, 14, and 15, which ranged from around 300 to 400 ms, and the last microstate are most probably tied to the LPP, even though local differences in parietal areas were not always present. There has been evidence of different spatial patterns of the LPP course, depending on valence and ER strategy^38^, and our results support that. We observed a drift of stronger positivity in the Active Viewing Positive condition from centro-parietal areas in the left hemisphere to frontal electrodes in the right hemisphere and back to the large centro-parietal cluster. In contrast, negative valence elicited stronger positivity over occipital areas. Surprisingly, there was also a lack of significant microstates between 400 and 500 ms, even though the LPP has its first peak anywhere between 250 and 600 ms^12,61^. This suggests that the LPP might not be a continuous phenomenon but an expression of different spatial distributions across time, akin to the three phase response to stimuli of different valence^62^. According to that study, the first effect of ER emerges after about 160 ms from prefrontal areas, the second at around 400 ms from the visual cortex, and the third at around 680 ms from the precentral gyrus. A good example of how much information can be retained when analyzing electrodes across the entire scalp.

Since positive and negative pictures were also less and more emotionally arousing, respectively, but the less and more emotionally arousing pictures were balanced in valence and showed no difference in microstates, there are three possible mechanisms causing differences here. The impact of valence is either independent of arousal, or there is an interaction between valence and arousal, or even a combination of the latter two mechanisms depending on the time point. An interaction has been suggested before^63–65^, but more research is needed to shine light on this.

#### Implications of the comparison of high and low emotional arousal

The lack of significant results in the emotional arousal-based comparison of the Active Viewing block was surprising, as we expected to see an indication of the EPN over temporo-occipital areas, which is associated with selective attention to highly emotionally arousing stimuli^16,66,67^. Especially since the median split of stimuli was successful in balancing valence between high and low emotional arousal, this lack of significance cannot be attributed to the qualities of the stimuli. Furthermore, the two significant microstate pairs in the study by Gianotti et al.^45^ had *p*-values higher than *p* = 0.01, so they would not have survived adjustment for multiple comparisons.

### Effects of ER strategies on different microstates

In the regular analysis, no strategy comparison yielded any significant differences. This was a very unexpected finding, since there is ample evidence of the impact of ER strategies on neural activity by ERP studies and fMRI studies alike^34,35,68–73^. However, sample sizes in such studies rarely exceed 30 participants, and the majority of ERP studies fail to report power calculations, including the relevant parameters for future sample size considerations^74^. It has been shown multiple times that behavioral research is overestimating reported effects and relations due to publication bias and problematic research practices^75,76^, and ERP studies are no exception. Furthermore, cognitive processes might not be comparable between those strategies in the first place, since they require very different mental operations^22,77^.

For Expressive Suppression, we expected to see differences similar to the N2 component^34^, to modulation of the posterior P1 and N170 components by facial muscles^78^, and in the time window of motor inhibition and response selection (250–350 ms) in frontal regions^17^. However, it is possible that motor inhibition was non-exclusive to Expressive Suppression due to a feeling of restriction from the EEG cap’s chin strap and the electrode placed below the participants’ left eye.

Since no microstate differences emerged from the repeated application of the ER strategies, the training effect might have been too small to detect, especially because it is smaller for down- than up-regulation^79^. Relating this finding to EEG studies of re-exposure^36,80^ is not appropriate because we presented new stimuli in the last block. Moreover, studies on ER training often either focus on behavioral outcomes or on improving ER by training a different skill altogether^81–84^.

The lack of differences regarding Detachment could be attributed to an interplay of three processes. First, participants might have (sub-)consciously applied reappraisal during some pictures in the Active Viewing block already, as that strategy is often chosen spontaneously in negative contexts^85^. Even though Active Viewing always preceded the ER blocks, the strategies might still have been readily accessible from the training. Secondly, the Detachment instruction was quite broad, so we cannot assume that the cognitive processes can easily be compared across participants or even trials. And lastly, the modulation of ERP amplitudes by reappraisal, especially between 200 and 300 ms, has been found to depend on subjective reappraisal success, suggesting there might not be a detectable difference if the regulation was successful^69^. However, raising these objections appears quite otiose, as there were differences between viewing and detaching from negative pictures in the exploratory analysis.

### Exploratory analysis

The exploratory analysis with the much larger time window of 600–2000 ms after stimulus onset yielded only three significant results across all contrasts, which were between Active Viewing and Detachment of negative pictures. One of these microstate pairs had a statistically significant result in the TANOVA, yet no significant results in the post-hoc tests, a phenomenon that had neither occurred in the regular analysis nor in the analysis by Gianotti et al.^45^. We therefore assume that the TANOVA is able to detect subtler deviations than an ERP analysis can, because of the way those subtle deviations accumulate to larger differences in the high-dimensional vector space.

While there was no difference between Active Viewing and Detachment of negative pictures in the regular analysis, it might have emerged in the exploratory one because the LPP in later stages does not depend on reappraisal success^69^. Both microstates two and four showed local differences with greater positivity for Detachment than for Active Viewing, which is in line with some LPP findings^34^ but not with others^42,86^. Furthermore, only the electrodes of microstate four resembled the common LPP recording site, while the three electrodes in microstate two were located in frontal and frontopolar areas. Activity in frontal areas during reappraisal is a common finding of ER studies^70,72,87,88^. Therefore, we cannot definitively say whether our ERP dependent findings corroborate these results, since Detachment elicited weaker instead of stronger amplitudes compared to Active Viewing. ER strategies rely on those prefrontal regions of cognitive control^89^ and a study using combined fMRI and EEG to investigate ER proposed an influence of prefrontal regions on the LPP^90^. The authors found that an accumulation of current density in posterior regions might directly or indirectly be influenced by the ventrolateral prefrontal cortex, reducing the LPP amplitude in reappraisal conditions. Furthermore, viewing highly emotionally arousing as opposed to neutral pictures has been shown to increase the similarity of frequencies in the prefrontal and parietal cortex in such a way that the prefrontal activity slightly precedes the posterior activity^91^. Since Detachment successfully reduced subjective emotional arousal ratings in the present study, we could translate this mechanism to Detachment and Active Viewing, with reduced activity in frontal and parietal regions indicating successful top-down regulation of emotion^92,93^. Building upon this, the fifth microstate, which showed no local differences, would be the residual effect of the top-down regulation with subthreshold differences between electrode pairs across the entire scalp.

Although the exploratory analysis yielded significant results for only one comparison of conditions, it was the one where differences in microstates were most likely, since reappraisal has been shown to have a stronger impact on the subjective emotional experience and its neural correlates than other ER strategies^23^. Additionally, the findings in the regular analysis were confined to the valence-based comparison, whereas the findings in the exploratory analysis were strategy-based. This illustrates the time scaling of perception and higher cognitive processes once more.

### Limitations and future directions

The results of the present study should be viewed in light of some limitations. First, the study was not designed for this replication, so pictures of positive and negative valence were confounded by emotional arousal. Second, the block design involved the risk of being in a strategy mindset beyond the stimulus duration but allowed deeper immersion into the strategy. Third, the lack of similar research made computational decisions and relations of results difficult. Fourth, ER strategies are quite complex and personal for direct comparison but we aimed for consensus by providing detailed instructions, training, and by asking the participants how they achieved each strategy after training and correcting their approach if necessary. Fifth, the expected training effect might be blurred since we do not know the participants’ motivation for choosing their strategy. And finally, we included all conditions into the grand–grandmean ERP computation, so differences between microstate pairs could just be latency differences of certain components^94^.

Despite these limitations, the present study has contributed to the establishment of microstate analysis for task-based paradigms. We preregistered all our analyses, and any deviation from Gianotti et al.^45^ was either deliberate to adhere to best practices of preprocessing or arose from vague descriptions in the original study. We showed that even a basal perceptual process like valence discrimination is neither locally nor temporally confined. This is certainly one explanation of why ERP studies often come to such seemingly incompatible findings. To avoid the disadvantages that arise from basing microstate analysis on ERPs, such as the general assumption of a stimulus-locked phase reset^95^, it would be feasible to use vectors of a time–frequency analysis instead, still benefitting from temporal and spatial resolution. Still, microstate analysis is in no way sufficient to determine the precise subcortical or even cortical sources of measured signals, so combined fMRI-EEG measuring or source modelling is definitely encouraged. Another interesting approach would be to invert the order of analysis, i.e. to calculate a TANOVA for every time point and define microstates based on successive time points with significant results^96^. However, this would require a powerful correction for multiple comparisons and a carefully designed paradigm. These approaches are just scratching the surface of possibilities for microstate analysis, and future investigations should apply these methods to a variety of task-based paradigms in a resourceful way. This can be done with the prospect of establishing a best practice of microstate computation, as well as fostering an analysis of EEG data beyond ERPs as the current common denominator.

### Methods

We report how we determined our sample size, all data exclusions (if any), all manipulations, and all measures in the study^97^. The data and analysis code as well as a list of all measures obtained is available in the Open Science Framework repository (https://osf.io/9auv5/). Only the study measures relevant to this analysis will be described below.

### Sample

Participants were recruited via the university’s central experimental ORSEE database^98^ and eligible if they stated that they had no current or past neurological/psychiatric disorders, no current use of medication or drugs that affected their performance (e.g. strong pain killers or marihuana), had normal/corrected vision, and were between 18 and 45 years old. The final sample consisted of *N* = 107 healthy adults (mean age 24.9 years, *SD* = 5.9, 67% female). Reverse power analysis using G*Power^99,100^ yielded an observable effect size of *d* = 0.27 for paired-sample t-tests, assuming a sample size of *N* = 107, a two-tailed significance level of *α* = 0.05, and a power of 1 − *β* = 0.80.

### Study design

Data had been collected as part of a larger project^101^, which was conducted in line with the Declaration of Helsinki and approved by the review board of the Dresden University of Technology (EK 286062019). Sessions were held between 9 a.m. and 5:30 p.m. Participants gave written informed consent, provided demographic data, and read the ER strategy instructions. For Active Viewing, participants were asked to “actively view all pictures and permit any emotions that may arise”. The instruction Expressive Suppression implied to allow emotions but omit facial reactions. Detachment was supposed to be achieved by distancing oneself from the picture, e.g. by imagining being a neutral observer. Participants practiced each strategy on 36 pictures. Then, a within-subject randomized block design was used to present emotional pictures on a computer screen, while recording physiological parameters using EEG, electrocardiogram, and eye tracking, and subjective ratings of emotional arousal. Each session lasted two to three hours, depending on ease of EEG preparation and the time participants needed to fill in several questionnaires after completing the task. The task itself took about 45 min and participants could take short breaks between blocks. Participants received eight euros per hour or course credit.

The paradigm was presented using *Presentation*^®^^102^ and consisted of a block of 25 neutral pictures, which we will not analyze, followed by four blocks of 50 emotional pictures each. The first larger block had the instruction Active Viewing, the second and third block had the instructions Detachment and Expressive Suppression, randomized between participants, and in the fourth block, Choice, participants could choose between Detachment and Expressive Suppression (Supplementary Figure S1).

### Stimuli

Pictures were taken from the Emotional Picture Set (EmoPicS)^103^ and the International Affective Picture System (IAPS)^104^. Their normative data was used to select 200 emotional pictures with extreme values on the valence scale and unambiguous content such as mutilation or laughing people. These 200 pictures were divided into four sets of 25 pictures with positive valence (*M* = 7.22, *SD* = 0.48, range 6.07–8.34, emotional arousal: *M* = 7.22, *SD* = 0.67, range 2.95–6.47) and four of 25 with negative valence (*M* = 4.87, *SD* = 0.57, range 1.31–3.77, emotional arousal: *M* = 6.20, *SD* = 0.73, range 4.93–7.99) (Supplementary Table S1). Positive pictures showed scenes like laughing families, kittens, or skydivers, negative pictures showed scenes like battered women, mutilation, or starving children. We used an evolutionary MATLAB^105^ algorithm to minimize the difference between means and standard deviations of sets of the same valence. One positive and one negative set were randomly paired for each block in every session. Separating pictures into high (*M* = 6.11, *SD* = 0.83, range 4.79–7.99, valence: *M* = 4.55, *SD* = 2.69, range 1.31–8.49) and low emotional arousal (*M* = 4.96, *SD* = 0.71, range 2.95–6.09, valence: *M* = 4.86, *SD* = 2.50, range 1.51–8.28) was achieved post hoc using a median split of the pictures of positive and negative valence, respectively (Supplementary Figure S2). Highly emotionally arousing stimuli showed scenes like execution, football fans, or erotic couples, less emotionally arousing stimuli showed scenes like injury, flowers, or playing children. T-tests revealed that this median split was successful in keeping valence balanced (Supplementary Figure S2). However, the pictures of positive and negative valence were confounded with emotional arousal, i.e. more negative pictures were also more emotionally arousing.

Each trial consisted of a black fixation cross on a grey background for 3000 ± 1000 ms, followed by the picture for 6000 ms, and a black screen for 1000 ms, resulting in a mean trial duration of 10,000 ms (Supplementary Figure S3). Pictures were randomized within each block. At the beginning of each block, the ER instruction appeared on screen. After each block, participants rated their subjective emotional arousal on a scale from “very low” to “very high” by moving a slider on screen with two keys, resulting in integers between − 200 and + 200. The ratings were used to ascertain the manipulation of eliciting emotions.

### Data acquisition and preprocessing

EEG was measured using a BrainCap with sintered Ag/AgCl sensors in the 10–20 system, sampled at 500 Hz using BrainAmp amplifiers (Brain Products GmbH, Gilching, Germany) and recorded with *BrainVision Recorder*^106^. Electrodes measured were the electrooculography electrode (EOG, below left eye), Fp1/2, Fz, F3/4, F7/8, F9/10, Cz, C1/2, C3/4, T7/8, CPz, CP1/2, CP3/4, Pz, P1/2, P3/4, P7/8, O1/2 and M2 (right mastoid). Channel AFz was used as ground; the online reference was the left mastoid. Skin was prepared with alcohol, abrasive paste, and electrolyte gel; all impedances were below 10 kΩ. Participants were seated in an electrically shielded cabin, while a chin rest kept the screen-eye-distance at around 60 cm. They were instructed to move as little as possible and blink only during the fixation cross and between blocks, when the experimenter checked in with them over intercom.

Preprocessing was done using *BrainVision Analyzer 2*^107^. Data were downsampled to 250 Hz and re-referenced to the mathematically linked mastoids, then filtered with zero phase shift Butterworth filters (2nd order low cutoff at 2 Hz, 4th order high cutoff at 40 Hz) and a 50 Hz notch filter. Following guidelines^108^, we deliberately deviated from the 2–20 Hz boxcar filter of Gianotti et al.^45^ as it smoothed the signal too severely. Data were then segmented into epochs of − 200 to + 600 ms after stimulus onset and baseline corrected using the pre-stimulus period. Segmented data were then subjected to manual artefact correction, with each 31-channel epoch displayed on full screen with a 20 μV y-axis. An epoch was excluded if it contained full or partial blinks (o-shaped distortions of the EOG), closed eyes (boxcar-shaped distortions of the EOG), muscular activity (sudden increase of noise), unassigned artefacts (large changes in single channels), and/or overall high noise. Finally, the EOG was excluded, the pre-stimulus period removed, and data, header, and marker files were exported into data files in multiplexed orientation using the American Standard Code for Information Interchange format.

### Microstate computation

**Figure 6:**
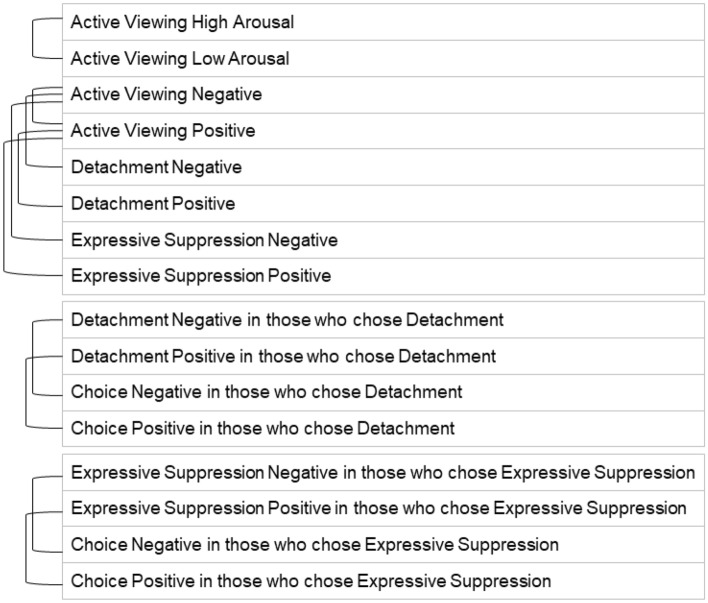
Conditions of interest and their comparisons. The upper eight conditions were computed by averaging the data across all participants. The lower eight conditions were computed by averaging the data of those participants who chose the same strategy in the last block. Brackets on the left indicate which conditions were compared with each other in the TANOVA.

Data files were loaded into *MATLAB*^105^ using the plugin *EEGLAB*^109^. 30-channel ERPs per subject were computed by averaging across epochs, and normalized by dividing all 30 values at each time point by the respective GFP. This ensures that any differences are not resulting from overall higher or lower activity, but from a change in activity distribution. The normalized ERPs were averaged across participants to obtain the grand–grandmean ERP, which was then subjected to *k*-means clustering to identify similar topographies. We computed *k* = 2 to *k* = 20, with 50 different centroid starting positions and maximum 100 iterations. We decided not to use 20 starting positions like Gianotti et al.^45^ did, because those did not yield robust results across multiple runs. The goal was to find a *k* with small inner-cluster-distances and yet no clusters that were too small to be considered a microstate. The optimal number was identified by plotting the sum of inner-cluster-distance measures against *k* in an elbow plot, and the durations of microstates for each *k* as a stacked bar chart (Supplementary Figures S4 and S5). Finally, a new 30-channel ERP for each of the 16 conditions of interest (Fig. 6) was computed and its GFP normalized. The number of microstates was applied to the ERPs by averaging across consecutive time points that belonged to a cluster.

### Statistical analysis

The manipulation check was computed using *RStudio*^110^, the *dplyr* package^111^, and the *DescTools* package^112^. An analysis of variance (ANOVA) was used to compare main effects of block (Active Viewing, Expressive Suppression, Detachment, Choice), valence (positive, negative), and their interaction on subjective emotional arousal ratings. Tukey’s Test was conducted for post-hoc comparisons.

Global microstate differences between conditions were analyzed with a topographical analysis of variance (TANOVA)^113^. For each hypothesis, the non-averaged data of two conditions A and B, i.e. positive versus negative pictures in Active Viewing, of all participants concerned were pooled and used for the computation of each microstate’s null distribution. The epochs within the data pool were shuffled randomly and the first *n* epochs, with *n* being the number of epochs of condition A in the data pool, were relabeled as condition A, the rest as condition B. The data within each newly labeled condition were averaged across epochs and normalized in their GFP. The resulting data were then averaged across those time points corresponding to a microstate, yielding as many 30-dimensional vectors as microstates in both conditions. Then, the angle measure *cos* *θ* was calculated between each vector pair, defined as:

$${\mathrm{cos}} \;\theta =\frac{\overrightarrow{A}\times \overrightarrow{B}}{\left|\overrightarrow{A}\right|\left|\overrightarrow{B}\right|}$$

The value of *cos* *θ* can range from − 1 to + 1, implying that the vectors are opposed or identical, respectively. Note that the denominator is referring to the vector’s Euclidean norm. The process of shuffling, relabeling, averaging, normalizing, and calculating was repeated 3000 times for each hypothesis^114^, resulting in a null distribution of 3000 values per microstate per hypothesis. The actual *cos* *θ* of every microstate pair was denoted as *cos* *θ*_MSx_, with *x* being the number of the microstate pair. The *p*-value for every microstate was then calculated by finding the rank position of the *cos* *θ*_MSx_ within the microstate’s sorted null distribution. To account for multiple comparisons, the significance level was adjusted by dividing *α* = 0.05 by the number of microstates, and microstate pairs with values of *p* < *α*_adj_ were defined as statistically significant.

Local differences between conditions were computed using two-sided paired-sample *t*-tests. For each hypothesis, data of conditions A and B were separately averaged across time points but not epochs into microstates. As many epochs as in the condition with less epochs were randomly selected from the other condition to meet the requirement for equally sized data. A *t*-test was then applied to every channel of every microstate that yielded *p* < *α*_adj_ in the TANOVA. To account for multiple comparisons, the significance level was adjusted by dividing α = 0.05 by the number of channels (*n* = 30), resulting in *α*_adj_ = 0.0017. Channels with values of *p* < *α*_adj_ (two-sided) were defined as statistically significant.

Descriptions of how each figure was processed are in Supplementary Table S6.

### Exploratory analysis

The epoch size of 600 ms was intended to enable comparison to Gianotti et al.^45^, but ER relies on cognitive processes beyond early perceptual components. For example, LPP differences between ER strategies start around 1000 ms after stimulus onset^35^. Therefore, the preprocessing steps and analyses were repeated with an epoch size of 600–2000 ms after stimulus onset. To retain marker information, epochs were shortened after data had been imported into *EEGLAB*. This exploratory analysis was already stated in our preregistration and did not depend on the results of the regular analysis.

## Supplementary Information

Supplementary Information.

## Data Availability

The data analyzed during this study, as well as preregistration, analysis code, and supplementary materials are freely available on the Open Science Framework repository (https://osf.io/9auv5/).
