# Supplementary material for: EEG microstate analysis of emotion regulation reveals no sequential processing of valence and emotional arousal
Source: Sci Rep. 2021 Oct 28;11:21277. doi: 10.1038/s41598-021-00731-7 (PMC8553854; doi:10.1038/s41598-021-00731-7)
Supplement: Supplementary file 1 — Supplementary Information. [file 41598_2021_731_MOESM1_ESM.pdf]

# Supplementary

**Title:**

EEG microstate analysis of emotion regulation reveals no sequential processing of valence and emotional arousal

**Authors:**

Zerna, Josephine[1]*, Strobel, Alexander[1], & Scheffel, Christoph[1]

**Affiliations:**

[1] Chair of Differential and Personality Psychology, Faculty of Psychology, Dresden University of Technology, Germany

* Corresponding Author: Josephine Zerna (josephine.zerna@tu-dresden.de)

**Journal:**

Nature Scientific Reports

**Supplementary Figure S1**

Block design of the emotion regulation paradigm. Participants were asked to view neutral and emotional pictures (Active Viewing condition), to omit all emotion related facial expressions (Expressive Suppression condition), and to distance themselves from the emotional content of the image (Detachment condition). Each participant was randomly assigned to one of the two possible block orders.

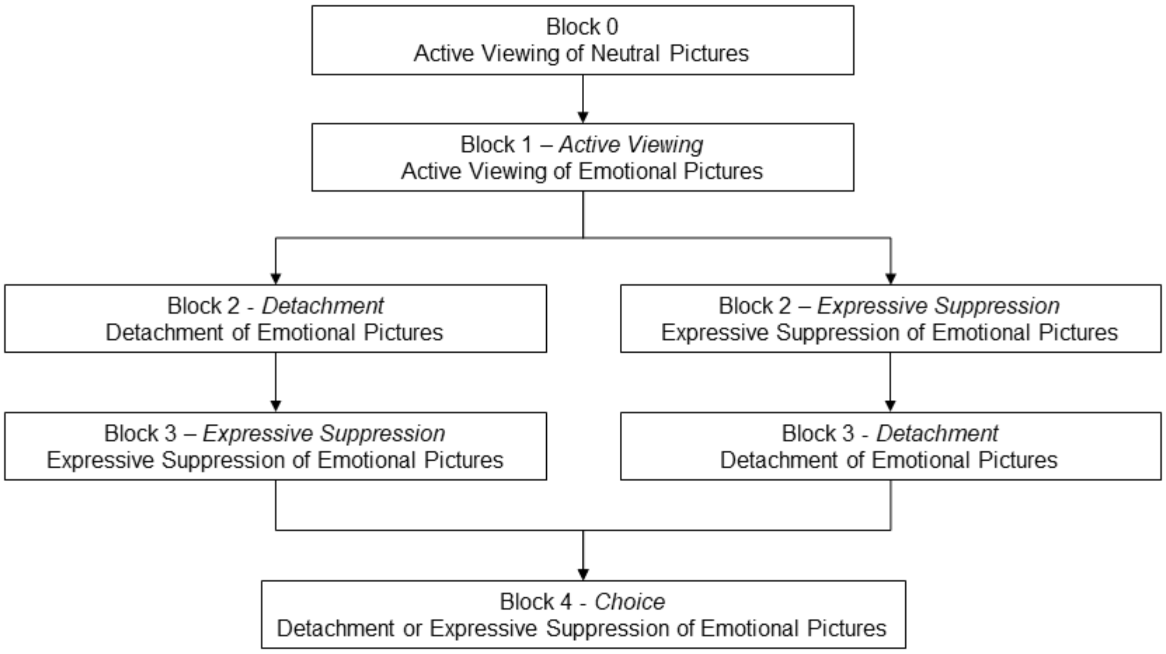

**Supplementary Figure S2**

Mean normative values and their SD (in brackets) of each subdivision of stimuli

| | | **Valence** | | | | |
|---|---|---|---|---|---|---|
| | | Low | High | | | |
| **Arousal** | Low | Valence 2.47 (0.58)<br>Arousal 5.58 (0.35) | Valence 7.25 (0.50)<br>Arousal 4.34 (0.32) | Valence 4.86 (2.46)<br>Arousal 4.96 (0.71) | N = 100 | Group means of valence were equal ($t(196) = 0.85$, $p = 0.39$), group means of arousal were not ($t(193) = 10.53$, $p < .001$) |
| | High | Valence 1.90 (0.41)<br>Arousal 6.81 (0.42) | Valence 7.19 (0.46)<br>Arousal 5.41 (0.46) | Valence 4.55 (2.69)<br>Arousal 6.11 (0.83) | N = 100 | |
| | | Valence 2.19 (0.57)<br>Arousal 6.20 (0.73) | Valence 7.22 (0.48)<br>Arousal 4.87 (0.67) | | | |
| | | N = 100 | N = 100 | | | |
| | | Group means of arousal ($t(192) = -67.29$, $p < .001$) and valence were not equal ($t(172) = -11.76$, $p < .001$) | | | | |

**Supplementary Figure S3**

Sequence of screens within each trial. Mean trial duration was 10 s. Example picture taken from the Open Affective Standardized Image Set OASIS [1].

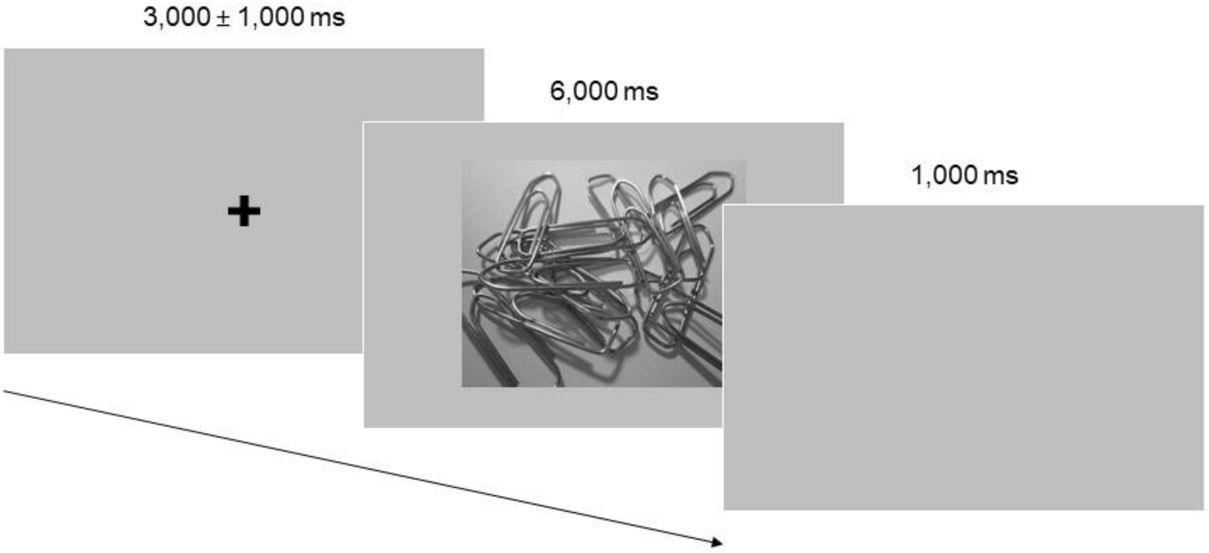

**Supplementary Figure S4**

Length of microstates in the clustering options $k$ = 2 to $k$ = 20

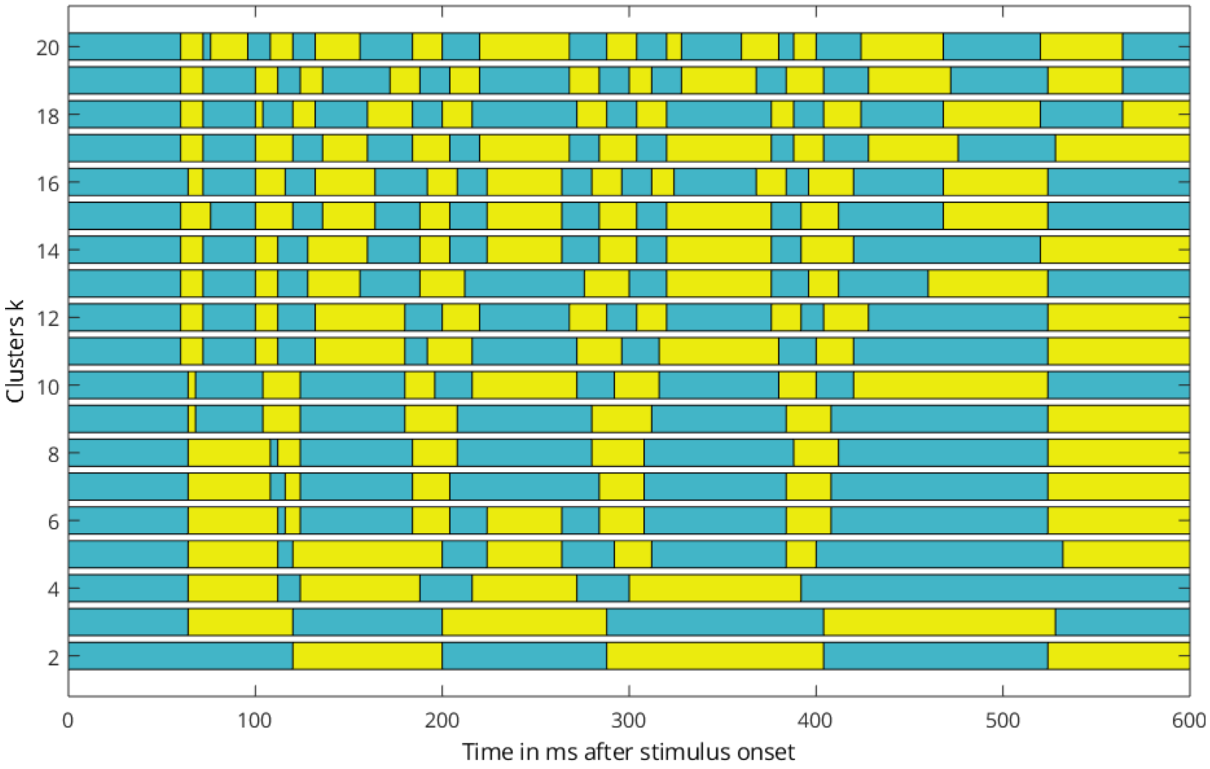

**Supplementary Figure S5**

Number of microstates (MS) (left panel) and sum of inner cluster distance measures in the clustering options $k$ = 2 to $k$ = 20 (right panel)

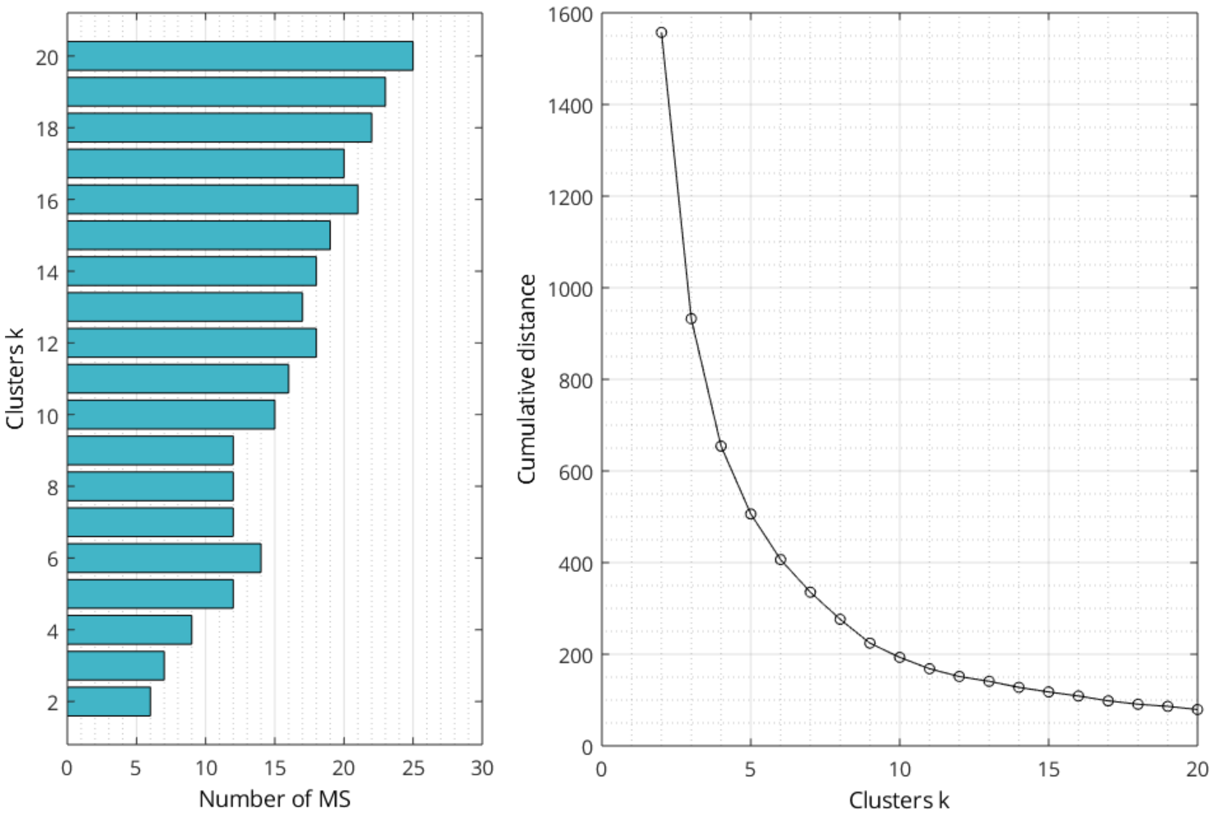

**Supplementary Figure S6**

Global and local microstate differences between actively viewing and detaching from negative pictures in the exploratory analysis (excerpt). Head seen from above, nose up. The first 18 of all 41 microstates are displayed. Time window is 600 ms to 1,272 ms after stimulus onset. Significant global differences indicated by * (padj < .001). Electrodes with local differences are plotted below the microstate pair, ▲ indicates higher values in Active Viewing Negative, ● indicates lower values in Detachment Negative, all padj < .0017. Colors range from -186 μV (teal) to +186 μV (yellow).

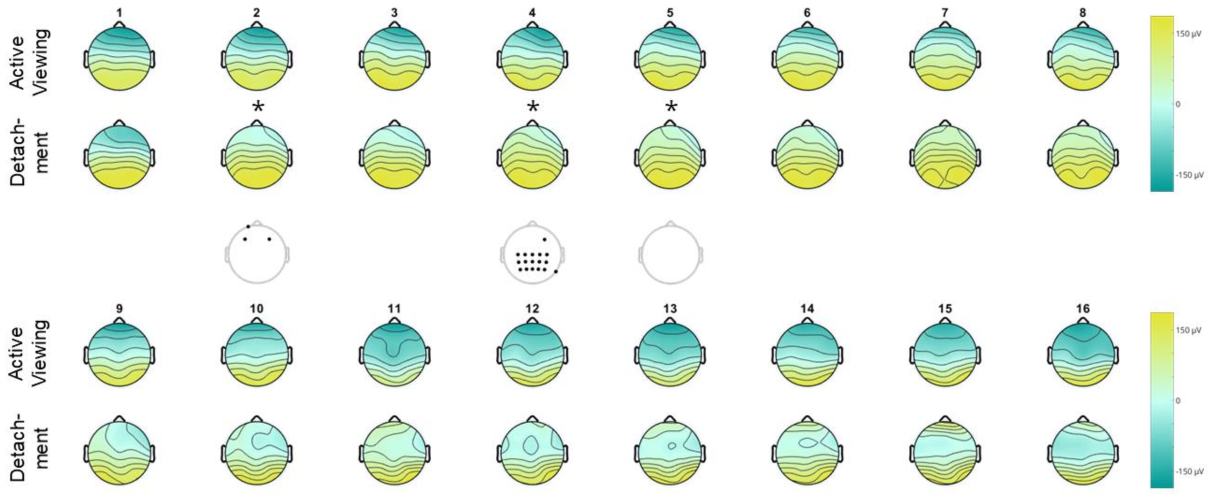

## Supplementary Table S1

List of IAPS [2] and EmoPicS [3] in each of the balanced sets.

| | Neutral | Positive 1 | Positive 2 | Positive 3 | Positive 4 | Negative 1 | Negative 2 | Negative 3 | Negative 4 |
|---|---|---|---|---|---|---|---|---|---|
| | 083† | 006† | 001† | 003† | 002† | 234† | 237† | 242† | 233† |
| | 107† | 011† | 004† | 023† | 005† | 248† | 239† | 252† | 236† |
| | 124† | 012† | 018† | 048† | 013† | 3068* | 240† | 3001* | 250† |
| | 140† | 021† | 022† | 050† | 026† | 3069* | 247† | 3010* | 1300* |
| | 143† | 029† | 024† | 051† | 028† | 3181* | 1111* | 3060* | 3005.1* |
| | 7000* | 032† | 043† | 058† | 037† | 3225* | 1280* | 3071* | 3053* |
| | 7001* | 039† | 063† | 073† | 052† | 3266* | 3000* | 3102* | 3080* |
| | 7002* | 053† | 078† | 077† | 074† | 3400* | 3015* | 3140* | 3168* |
| | 7003* | 054† | 260† | 331† | 075† | 3550* | 3030* | 6022* | 3170* |
| | 7004* | 055† | 262† | 1340* | 258† | 6350* | 3051* | 6831* | 3350* |
| | 7006* | 057† | 1440* | 1630* | 1610* | 6520* | 3063* | 8230* | 3530* |
| | 7009* | 256† | 1441* | 1750* | 1710* | 8485* | 3150* | 8231* | 6212* |
| | 7017* | 257† | 1595* | 1920* | 1721* | 9040* | 3180* | 9320* | 6415* |
| | 7021* | 1410* | 1659* | 2019* | 2224* | 9140* | 3301* | 9410* | 9250* |
| | 7025* | 1463* | 2071* | 2030* | 2345* | 9163* | 6313* | 9420* | 9253* |
| | 7041* | 1601* | 2091* | 2045* | 2660* | 9181* | 6550* | 9423* | 9373* |
| | 7100* | 2274* | 2151* | 2070* | 4150* | 9182* | 7361* | 9427* | 9405* |
| | 7150* | 2314* | 2155* | 2092* | 4532* | 9183* | 9300* | 9428* | 9421* |
| | 7185* | 2347* | 2311* | 2165* | 4542* | 9325* | 9332* | 9430* | 9425* |
| | 7186* | 4599* | 4250* | 2550* | 4574* | 9414* | 9400* | 9500* | 9433* |
| | 7187* | 4628* | 4607* | 4597* | 4608* | 9419* | 9412* | 9571* | 9490* |
| | 7211* | 4653* | 5210* | 4612* | 4614* | 9424* | 9413* | 9800* | 9520* |
| | 7224* | 7330* | 7660* | 4651* | 4641* | 9480* | 9530* | 9908* | 9560* |
| | 7233* | 7472* | 8120* | 5833* | 5825* | 9635.1* | 9570* | 9910* | 9599* |
| | 7235* | 8496* | 8500* | 7230* | 8461* | 9904* | 9902* | 9940* | 9921* |
| **Valence** | 4.86 ± 0.49 | 7.22 ± 0.34 | 7.22 ± 0.50 | 7.22 ± 0.60 | 7.22 ± 0.49 | 2.18 ± 0.53 | 2.19 ± 0.58 | 2.19 ± 0.56 | 2.19 ± 0.65 |
| **Arousal** | 3.01 ± 0.61 | 4.87 ± 0.71 | 4.87 ± 0.63 | 4.87 ± 0.73 | 4.87 ± 0.62 | 6.20 ± 0.70 | 6.20 ± 0.79 | 6.20 ± 0.75 | 6.20 ± 0.72 |

Note. * Pictures taken from the IAPS [2]; † Pictures taken from the EmoPicS [3].

**Supplementary Table S2**

Duration (in ms) and time window (in ms after stimulus onset (SO)) of every microstate (MS) in the regular analysis

| MS | 1 | 2 | 3 | 4 | 5 | 6 |
|---|---|---|---|---|---|---|
| Time points | 15 | 3 | 7 | 3 | 4 | 8 |
| Duration | 60 | 12 | 28 | 12 | 16 | 32 |
| Time after SO | 0-60 | 60-72 | 72-100 | 100-112 | 112-128 | 128-160 |
| Cluster ID | 2 | 11 | 6 | 11 | 9 | 12 |
| MS | 7 | 8 | 9 | 10 | 11 | 12 |
| Time points | 7 | 4 | 5 | 10 | 5 | 5 |
| Duration | 28 | 16 | 20 | 40 | 20 | 20 |
| Time after SO | 160-188 | 188-204 | 204-224 | 224-264 | 264-284 | 284-304 |
| Cluster ID | 3 | 5 | 13 | 1 | 13 | 5 |
| MS | 13 | 14 | 15 | 16 | 17 | 18 |
| Time points | 4 | 14 | 4 | 7 | 25 | 20 |
| Duration | 16 | 56 | 16 | 28 | 100 | 80 |
| Time after SO | 304-320 | 320-376 | 376-392 | 392-420 | 420-520 | 520-600 |
| Cluster ID | 10 | 4 | 10 | 14 | 7 | 8 |

## Supplementary Table S3

Results of the two-sided paired-sample t-tests for the microstates (MS) with significant global differences in the regular analysis in the comparison of Active Viewing Positive and Active Viewing Negative. First row indicates the microstate number, columns for each microstate indicate significance for padj = .0028 (1 = significant, 0 = not significant), p-value and t-value with 106 degrees of freedom.

| **MS** | **3** | | | **6** | | | **7** | | | **13** | | |
|---|---|---|---|---|---|---|---|---|---|---|---|---|
| **Channel** | **Sign** | **p** | **t** | **Sign** | **p** | **t** | **Sign** | **p** | **t** | **Sign** | **p** | **t** |
| Fp1 | 0 | 0,0046 | -2,838 | 0 | 0,0221 | 2,290 | 1 | 8,42E-05 | 3,942 | 0 | 0,6874 | 0,402 |
| Fp2 | 1 | 0,0008 | -3,374 | 0 | 0,0540 | 1,928 | 1 | 0,0002 | 3,683 | 0 | 0,4445 | 0,765 |
| F9 | 0 | 0,0326 | -2,138 | 1 | 9,85E-05 | 3,904 | 1 | 1,81E-10 | 6,418 | 0 | 0,1705 | 1,371 |
| F7 | 0 | 0,0475 | -1,983 | 1 | 0,0002 | 3,762 | 1 | 6,70E-10 | 6,210 | 0 | 0,0594 | 1,887 |
| F3 | 0 | 0,0667 | -1,835 | 1 | 0,0001 | 3,852 | 1 | 2,16E-07 | 5,207 | 1 | 0,0002 | 3,786 |
| Fz | 0 | 0,0656 | -1,842 | 1 | 0,0001 | 3,877 | 1 | 1,29E-06 | 4,859 | 1 | 4,03E-05 | 4,117 |
| F4 | 0 | 0,0154 | -2,425 | 1 | 7,51E-05 | 3,970 | 1 | 3,83E-06 | 4,636 | 1 | 0,0001 | 3,806 |
| F8 | 0 | 0,0156 | -2,421 | 1 | 0,0002 | 3,739 | 1 | 0,0010 | 3,283 | 0 | 0,0253 | 2,238 |
| F10 | 0 | 0,0422 | -2,033 | 0 | 0,0025 | 3,031 | 0 | 0,0501 | 1,961 | 0 | 0,1227 | 1,544 |
| T7 | 0 | 0,7722 | -0,290 | 1 | 4,80E-06 | 4,589 | 1 | 7,70E-07 | 4,962 | 1 | 0,0002 | 3,751 |
| C3 | 0 | 0,7810 | -0,278 | 1 | 2,60E-06 | 4,716 | 1 | 6,19E-08 | 5,438 | 1 | 7,93E-06 | 4,481 |
| C1 | 0 | 0,9468 | 0,067 | 1 | 5,34E-06 | 4,566 | 1 | 1,06E-07 | 5,340 | 1 | 3,05E-06 | 4,683 |
| Cz | 0 | 0,9466 | 0,067 | 1 | 1,22E-05 | 4,387 | 1 | 5,14E-07 | 5,041 | 1 | 2,74E-06 | 4,706 |
| C2 | 0 | 0,8935 | 0,134 | 1 | 1,36E-06 | 4,848 | 1 | 3,54E-07 | 5,113 | 1 | 7,79E-06 | 4,485 |
| C4 | 0 | 0,9657 | 0,043 | 1 | 2,59E-07 | 5,173 | 1 | 5,97E-07 | 5,012 | 1 | 8,73E-05 | 3,933 |
| T8 | 0 | 0,5521 | -0,595 | 1 | 7,96E-07 | 4,956 | 1 | 0,0006 | 3,456 | 0 | 0,0291 | 2,184 |
| CP3 | 0 | 0,3563 | 0,923 | 1 | 4,93E-07 | 5,049 | 1 | 1,88E-07 | 5,234 | 1 | 4,50E-05 | 4,091 |
| CP1 | 0 | 0,3560 | 0,923 | 1 | 1,53E-06 | 4,825 | 1 | 8,03E-08 | 5,391 | 1 | 8,41E-05 | 3,942 |
| CPz | 0 | 0,3595 | 0,917 | 1 | 1,33E-06 | 4,853 | 1 | 9,20E-08 | 5,366 | 1 | 0,0002 | 3,716 |
| CP2 | 0 | 0,2752 | 1,091 | 1 | 3,26E-07 | 5,129 | 1 | 5,71E-07 | 5,021 | 1 | 0,0005 | 3,485 |
| CP4 | 0 | 0,1977 | 1,289 | 1 | 3,09E-07 | 5,139 | 1 | 7,90E-06 | 4,482 | 0 | 0,0022 | 3,073 |
| P7 | 0 | 0,0173 | 2,382 | 0 | 0,2286 | 1,204 | 0 | 0,7075 | -0,375 | 1 | 0,0004 | 3,564 |
| P3 | 0 | 0,0765 | 1,772 | 1 | 3,92E-05 | 4,123 | 1 | 0,0004 | 3,570 | 0 | 0,0021 | 3,080 |
| P1 | 0 | 0,1211 | 1,551 | 1 | 1,98E-06 | 4,772 | 1 | 5,74E-06 | 4,551 | 0 | 0,0030 | 2,973 |
| Pz | 0 | 0,1233 | 1,542 | 1 | 2,65E-06 | 4,713 | 1 | 3,94E-06 | 4,630 | 0 | 0,0122 | 2,510 |
| P2 | 0 | 0,1003 | 1,644 | 1 | 6,11E-06 | 4,537 | 1 | 3,26E-05 | 4,166 | 0 | 0,0195 | 2,338 |
| P4 | 0 | 0,0673 | 1,831 | 1 | 6,77E-05 | 3,994 | 0 | 0,0029 | 2,983 | 0 | 0,0300 | 2,172 |
| P8 | 0 | 0,0024 | 3,038 | 0 | 0,6328 | -0,478 | 0 | 0,0045 | -2,846 | 0 | 0,1907 | 1,309 |
| O1 | 0 | 0,0202 | 2,325 | 0 | 0,7662 | -0,297 | 0 | 0,1472 | -1,450 | 0 | 0,3029 | 1,030 |
| O2 | 0 | 0,0312 | 2,157 | 0 | 0,4999 | -0,675 | 0 | 0,0547 | -1,922 | 0 | 0,5891 | -0,540 |

## Supplementary Table S3 (continuation)

| MS | 14 | | | 15 | | | 18 | | |
|---|---|---|---|---|---|---|---|---|---|
| Channel | Sign | p | t | Sign | p | t | Sign | p | t |
| Fp1 | 0 | 0,0019 | 3,104 | 0 | 0,0151 | 2,432 | 0 | 0,0634 | 1,857 |
| Fp2 | 1 | 0,0004 | 3,560 | 0 | 0,0026 | 3,014 | 0 | 0,0041 | 2,876 |
| F9 | 0 | 0,0069 | 2,704 | 0 | 0,0169 | 2,392 | 0 | 0,6096 | -0,511 |
| F7 | 1 | 6,50E-05 | 4,004 | 0 | 0,0022 | 3,063 | 0 | 0,3598 | 0,916 |
| F3 | 1 | 3,73E-07 | 5,103 | 1 | 0,0004 | 3,530 | 1 | 0,0006 | 3,449 |
| Fz | 1 | 1,36E-08 | 5,707 | 1 | 0,0002 | 3,732 | 1 | 5,25E-05 | 4,055 |
| F4 | 1 | 8,56E-08 | 5,379 | 0 | 0,0030 | 2,974 | 1 | 8,67E-06 | 4,462 |
| F8 | 1 | 2,02E-09 | 6,030 | 1 | 4,57E-05 | 4,087 | 1 | 0,0013 | 3,221 |
| F10 | 1 | 1,28E-07 | 5,305 | 1 | 2,30E-05 | 4,246 | 0 | 0,0100 | 2,579 |
| T7 | 1 | 4,63E-05 | 4,084 | 0 | 0,0758 | 1,776 | 0 | 0,8467 | 0,193 |
| C3 | 1 | 1,38E-06 | 4,846 | 0 | 0,0754 | 1,779 | 0 | 0,0023 | 3,054 |
| C1 | 1 | 2,71E-07 | 5,164 | 0 | 0,0336 | 2,126 | 1 | 7,47E-06 | 4,494 |
| Cz | 1 | 3,04E-07 | 5,142 | 0 | 0,0469 | 1,988 | 1 | 1,37E-06 | 4,848 |
| C2 | 1 | 7,81E-07 | 4,959 | 0 | 0,0258 | 2,231 | 1 | 2,36E-06 | 4,737 |
| C4 | 1 | 2,63E-07 | 5,170 | 0 | 0,0151 | 2,432 | 1 | 5,62E-05 | 4,039 |
| T8 | 1 | 7,34E-06 | 4,498 | 0 | 0,0288 | 2,187 | 0 | 0,0281 | 2,198 |
| CP3 | 1 | 0,0005 | 3,467 | 0 | 0,3711 | 0,895 | 0 | 0,0036 | 2,912 |
| CP1 | 1 | 0,0003 | 3,649 | 0 | 0,3664 | 0,903 | 1 | 5,84E-05 | 4,030 |
| CPz | 1 | 0,0002 | 3,745 | 0 | 0,3863 | 0,867 | 1 | 5,02E-06 | 4,579 |
| CP2 | 1 | 0,0002 | 3,686 | 0 | 0,3684 | 0,900 | 1 | 2,06E-05 | 4,271 |
| CP4 | 1 | 0,0003 | 3,585 | 0 | 0,3216 | 0,991 | 1 | 0,0003 | 3,630 |
| P7 | 0 | 0,9595 | -0,051 | 0 | 0,1759 | -1,354 | 0 | 0,6876 | -0,402 |
| P3 | 0 | 0,3039 | 1,028 | 0 | 0,5958 | -0,531 | 0 | 0,0279 | 2,200 |
| P1 | 0 | 0,0989 | 1,651 | 0 | 0,8048 | -0,247 | 0 | 0,0041 | 2,872 |
| Pz | 0 | 0,1656 | 1,387 | 0 | 0,6470 | -0,458 | 1 | 0,0012 | 3,244 |
| P2 | 0 | 0,2248 | 1,214 | 0 | 0,4552 | -0,747 | 1 | 0,0012 | 3,237 |
| P4 | 0 | 0,4524 | 0,752 | 0 | 0,2190 | -1,230 | 0 | 0,0102 | 2,572 |
| P8 | 0 | 0,3629 | -0,910 | 0 | 0,0115 | -2,531 | 0 | 0,6391 | 0,469 |
| O1 | 1 | 1,40E-05 | -4,357 | 1 | 0,0005 | -3,479 | 0 | 0,9444 | -0,070 |
| O2 | 1 | 5,84E-07 | -5,016 | 1 | 2,31E-05 | -4,245 | 0 | 0,8052 | 0,247 |

## Supplementary Table S4

Duration (in ms) and time window (in ms after stimulus onset (SO)) of every microstate (MS) in the exploratory analysis

| MS | 1 | 2 | 3 | 4 | 5 | 6 |
|---|---|---|---|---|---|---|
| Time points | 11 | 6 | 16 | 2 | 3 | 9 |
| Duration | 44 | 24 | 64 | 8 | 12 | 36 |
| Time after SO | 600-644 | 644-668 | 668-732 | 732-740 | 740-752 | 752-788 |
| Cluster | 12 | 7 | 6 | 7 | 6 | 7 |
| MS | 7 | 8 | 9 | 10 | 11 | 12 |
| Time points | 5 | 5 | 16 | 14 | 11 | 2 |
| Duration | 20 | 20 | 64 | 56 | 44 | 8 |
| Time after SO | 788-808 | 808-828 | 828-892 | 892-948 | 948-992 | 992-1000 |
| Cluster | 6 | 7 | 4 | 10 | 5 | 14 |
| MS | 13 | 14 | 15 | 16 | 17 | 18 |
| Time points | 20 | 12 | 9 | 8 | 8 | 11 |
| Duration | 80 | 48 | 36 | 32 | 32 | 44 |
| Time after SO | 1000-1080 | 1080-1128 | 1128-1164 | 1164-1196 | 1196-1228 | 1228-1272 |
| Cluster | 3 | 13 | 3 | 14 | 13 | 14 |
| MS | 19 | 20 | 21 | 22 | 23 | 24 |
| Time points | 3 | 3 | 5 | 3 | 12 | 11 |
| Duration | 12 | 12 | 20 | 12 | 48 | 44 |
| Time after SO | 1272-1284 | 1284-1296 | 1296-1216 | 1316-1328 | 1328-1376 | 1376-1420 |
| Cluster | 3 | 13 | 14 | 13 | 14 | 2 |
| MS | 25 | 26 | 27 | 28 | 29 | 30 |
| Time points | 6 | 2 | 13 | 20 | 4 | 1 |
| Duration | 24 | 8 | 52 | 80 | 16 | 4 |
| Time after SO | 1420-1444 | 1444-1452 | 1452-1504 | 1504-1584 | 1584-1600 | 1600-1604 |
| Cluster | 8 | 2 | 9 | 2 | 8 | 13 |
| MS | 31 | 32 | 33 | 34 | 35 | 36 |
| Time points | 22 | 4 | 4 | 9 | 4 | 2 |
| Duration | 88 | 16 | 16 | 36 | 16 | 8 |
| Time after SO | 1604-1692 | 1692-1708 | 1708-1724 | 1724-1760 | 1760-1776 | 1776-1784 |
| Cluster | 8 | 2 | 8 | 11 | 8 | 11 |
| MS | 37 | 38 | 39 | 40 | 41 | |
| Time points | 4 | 5 | 7 | 16 | 22 | |
| Duration | 16 | 20 | 28 | 64 | 88 | |
| Time after SO | 1784-1800 | 1800-1820 | 1820-1848 | 1848-1912 | 1912-2000 | |
| Cluster | 9 | 11 | 9 | 11 | 1 | |

## Supplementary Table S5

Results of the two-sided paired-sample *t*-tests for the microstates (MS) with significant global differences in the exploratory analysis in the comparison of Active Viewing Negative and Detachment Negative. First row indicates the microstate number, columns for each microstate indicate significance for $p_{adj}$ = .0012 (1 = significant, 0 = not significant), p-value and t-value with 106 degrees of freedom.

| **MS** | **2** | | | **4** | | | **5** | | |
|---|---|---|---|---|---|---|---|---|---|
| **Channel** | **Sign** | **p** | **t** | **Sign** | **p** | **t** | **Sign** | **p** | **t** |
| Fp1 | 1 | 0,0012 | -3,241 | 0 | 0,0300 | -2,172 | 0 | 0,0025 | -3,028 |
| Fp2 | 0 | 0,0024 | -3,046 | 0 | 0,0105 | -2,563 | 0 | 0,0056 | -2,772 |
| F9 | 0 | 0,1480 | -1,447 | 0 | 0,1935 | -1,301 | 0 | 0,3635 | -0,909 |
| F7 | 0 | 0,0364 | -2,094 | 0 | 0,0265 | -2,221 | 0 | 0,0086 | -2,630 |
| F3 | 1 | 0,0015 | -3,174 | 0 | 0,0022 | -3,065 | 0 | 0,0223 | -2,288 |
| Fz | 0 | 0,0029 | -2,987 | 0 | 0,0029 | -2,984 | 0 | 0,0193 | -2,342 |
| F4 | 1 | 0,0012 | -3,250 | 1 | 0,0007 | -3,397 | 0 | 0,0029 | -2,977 |
| F8 | 0 | 0,0174 | -2,381 | 0 | 0,0346 | -2,114 | 0 | 0,0857 | -1,719 |
| F10 | 0 | 0,2120 | -1,249 | 0 | 0,4971 | -0,679 | 0 | 0,7188 | -0,360 |
| T7 | 0 | 0,0505 | -1,957 | 0 | 0,0097 | -2,589 | 0 | 0,1200 | -1,555 |
| C3 | 0 | 0,0036 | -2,916 | 1 | 0,0003 | -3,629 | 0 | 0,0215 | -2,301 |
| C1 | 0 | 0,0024 | -3,037 | 1 | 0,0003 | -3,658 | 0 | 0,0112 | -2,539 |
| Cz | 0 | 0,0028 | -2,992 | 1 | 0,0005 | -3,502 | 0 | 0,0105 | -2,561 |
| C2 | 0 | 0,0031 | -2,959 | 1 | 0,0005 | -3,514 | 0 | 0,0066 | -2,722 |
| C4 | 0 | 0,0051 | -2,803 | 1 | 0,0010 | -3,302 | 0 | 0,0043 | -2,857 |
| T8 | 0 | 0,0018 | -3,132 | 0 | 0,0149 | -2,438 | 0 | 0,3195 | -0,996 |
| CP3 | 0 | 0,0097 | -2,591 | 1 | 0,0002 | -3,783 | 0 | 0,0087 | -2,625 |
| CP1 | 0 | 0,0031 | -2,959 | 1 | 0,0002 | -3,761 | 0 | 0,0066 | -2,720 |
| CPz | 0 | 0,0037 | -2,906 | 1 | 0,0003 | -3,591 | 0 | 0,0063 | -2,734 |
| CP2 | 0 | 0,0036 | -2,914 | 1 | 0,0004 | -3,523 | 0 | 0,0054 | -2,787 |
| CP4 | 0 | 0,0049 | -2,815 | 1 | 0,0010 | -3,288 | 0 | 0,0066 | -2,718 |
| P7 | 0 | 0,1021 | -1,636 | 0 | 0,0030 | -2,972 | 0 | 0,0426 | -2,029 |
| P3 | 0 | 0,0136 | -2,471 | 1 | 0,0001 | -3,851 | 0 | 0,0043 | -2,856 |
| P1 | 0 | 0,0042 | -2,870 | 1 | 0,0002 | -3,758 | 0 | 0,0058 | -2,764 |
| Pz | 0 | 0,0043 | -2,862 | 1 | 0,0005 | -3,468 | 0 | 0,0071 | -2,694 |
| P2 | 0 | 0,0039 | -2,887 | 1 | 0,0006 | -3,451 | 0 | 0,0059 | -2,755 |
| P4 | 0 | 0,0041 | -2,871 | 1 | 0,0008 | -3,345 | 0 | 0,0044 | -2,851 |
| P8 | 0 | 0,0357 | -2,102 | 1 | 0,0013 | -3,225 | 0 | 0,0100 | -2,580 |
| O1 | 0 | 0,0037 | -2,907 | 0 | 0,0020 | -3,099 | 0 | 0,0102 | -2,573 |
| O2 | 0 | 0,0030 | -2,971 | 0 | 0,0086 | -2,629 | 0 | 0,0175 | -2,378 |

## Supplementary Table S6

Description of how each figure in the manuscript was processed.

| Figure | Process |
|---|---|
| 1 | The left and right panel were created separately in *R Studio*, brackets and asterisks indicating significance were added in *Microsoft Power Point* |
| 2 | Entirely created in *Microsoft Power Point* |
| 3 | Entirely created in *Microsoft Power Point* |
| 4 | The topoplots and the colour bars were created separately in *MATLAB* using *EEGLAB*, condition names and asterisks indicating significance were added in *Microsoft Power Point* |
| 5 | The topoplots and the colour bars were created separately in *MATLAB* using *EEGLAB*, condition names and asterisks indicating significance were added in *Microsoft Power Point* |
| 6 | Entirely created in *Microsoft Power Point* |

## References


1. Kurdi, B., Lozano, S. & Banaji, M. R. Introducing the Open Affective Standardized Image Set (OASIS). *Behav. Res. Methods* **49**, 457–470 (2017).
2. Lang, P. J., Bradley, M. M. & Cuthbert, B. N. *International Affective Picture System (IAPS): Affective ratings of pictures and instruction manual.* (University of Florida, 2008).
3. Wessa, M. *et al.* EmoPics: Subjektive und psychophysiologische Evaluationen neuen Bildmaterials für die klinisch-bio-psychologische Forschung. *Z Klin Psychol Psychother* **1**, S11–S77 (2010).
